# The utility of behavioral biometrics in user authentication and demographic characteristic detection: a scoping review

**DOI:** 10.1186/s13643-024-02451-1

**Published:** 2024-02-08

**Authors:** O. L. Finnegan, J. W. White, B. Armstrong, E. L. Adams, S. Burkart, M. W. Beets, S. Nelakuditi, E. A. Willis, L. von Klinggraeff, H. Parker, M. Bastyr, X. Zhu, Z. Zhong, R. G. Weaver

**Affiliations:** 1 Department of Exercise Science, University of South Carolina, Columbia, USA; 2 Department of Computer Science and Engineering, University of South Carolina, Columbia, USA; 3 Center for Health Promotion and Disease Prevention, University of North Carolina Chapel Hill, Chapel Hill, USA

## Abstract

**Background:**

Objective measures of screen time are necessary to better understand the complex relationship between screen time and health outcomes. However, current objective measures of screen time (e.g., passive sensing applications) are limited in identifying the user of the mobile device, a critical limitation in children’s screen time research where devices are often shared across a family. Behavioral biometrics, a technology that uses embedded sensors on modern mobile devices to continuously authenticate users, could be used to address this limitation.

**Objective:**

The purpose of this scoping review was to summarize the current state of behavioral biometric authentication and synthesize these findings within the scope of applying behavioral biometric technology to screen time measurement.

**Methods:**

We systematically searched five databases (Web of Science Core Collection, Inspec in Engineering Village, Applied Science & Technology Source, IEEE Xplore, PubMed), with the last search in September of 2022. Eligible studies were on the authentication of the user or the detection of demographic characteristics (age, gender) using built-in sensors on mobile devices (e.g., smartphone, tablet). Studies were required to use the following methods for authentication: motion behavior, touch, keystroke dynamics, and/or behavior profiling. We extracted study characteristics (sample size, age, gender), data collection methods, data stream, model evaluation metrics, and performance of models, and additionally performed a study quality assessment. Summary characteristics were tabulated and compiled in Excel. We synthesized the extracted information using a narrative approach.

**Results:**

Of the 14,179 articles screened, 122 were included in this scoping review. Of the 122 included studies, the most highly used biometric methods were touch gestures (*n* = 76) and movement (*n* = 63), with 30 studies using keystroke dynamics and 6 studies using behavior profiling. Of the studies that reported age (47), most were performed exclusively in adult populations (*n* = 34). The overall study quality was low, with an average score of 5.5/14.

**Conclusion:**

The field of behavioral biometrics is limited by the low overall quality of studies. Behavioral biometric technology has the potential to be used in a public health context to address the limitations of current measures of screen time; however, more rigorous research must be performed in child populations first.

**Systematic review registration:**

The protocol has been pre-registered in the Open Science Framework database (10.17605/OSF.IO/92YCT).

**Supplementary Information:**

The online version contains supplementary material available at 10.1186/s13643-024-02451-1.

## Introduction

Screen time is a critical health behavior related to a variety of health outcomes in children [1–6]. Historically, measuring screen time has been reliant on self-report or proxy-report measures [7], due in part to the nature of digital media consumption (e.g., in-home computer and TV use). The introduction of mobile devices (tablets, smartphones) has substantially altered the landscape of digital media consumption, and these devices have become the favored media choice for children due to their portability, interactivity, and capacity to stream a wide range of content [8–11]. Self-report measures are of limited validity in assessing mobile screen use due to the intermittent and on-demand use of mobile devices, which hamper one’s ability to retrospectively report screen time [7, 12, 13]. In addition to not being sensitive enough to sufficiently capture all mobile screen use, self-report measures are also subject to recall bias and social desirability bias [14, 15]. Given the proliferation of mobile devices [8, 10], there has been a growing demand to advance our current screen time measures to more effectively capture mobile screen use [16], specifically using objective measures [17].

Researchers have begun to use passive sensing applications (e.g., Chronicle) to overcome the limitations of subjective reports and which unobtrusively monitor mobile screen use on mobile devices [13, 18]. Chronicle is an Android passive sensing application that tracks the duration, frequency, and timing of data, general application type, and application status (foreground vs. background) using Google API every 15 s [13]. Benefits of passive sensing applications include a reduced researcher and participant burden compared to self-report measures and lower cost for researchers to employ. However, while this data can be relevant for tracking the duration of use and the context of use, these passive sensing applications are not able to capture who specifically is using the device. For child screen time research, this limitation in identifying the user of a device is of particular concern as mobile devices are often shared between siblings or between the parent and the child [12, 19]. Therefore, identifying the user of the device is critical to optimizing the potential for passive sensing methods in tracking objective screen use metrics in children.

Behavioral biometrics could be used to address this shortcoming of objective screen time measurement by identifying users of mobile devices. Modern mobile devices contain a variety of sensors (e.g., accelerometer, gyroscope, magnetometer, touch) that collect multiple data streams and can provide characteristic information about the user. These sensors provide the basis for behavioral biometric authentication [20–22]. Unlike physiological biometrics (e.g., fingerprint, iris, facial recognition), behavioral biometrics do not require additional hardware in modern mobile devices [23, 24], making it a feasible research tactic for screen time measurement. Additionally, behavioral biometrics can provide continuous user authentication, whereas physiological biometrics are typically a one-time authentication for gaining access to a device [23]. There are several types of behavioral biometrics used for authentication, including behavior profiling, keystroke dynamics (typing dynamics), touch dynamics, and motion behavior [23]. Behavior profiling uses data such as the type of applications being used and battery life (host-based approach) as well as calls, texts, and GPS location (network-based approach) for user authentication [21]. This type of authentication has been used for fraud detection systems, in which unusual activities (e.g., calls, texts) and a new location can identify device theft and subsequently initiate a fraud protection mechanism [25]. Keystroke dynamics involves the characteristic way in which an individual types, specifically identifying the habitual typing pattern [21]. There are two types of keystroke dynamics, including static text, which analyzes a fixed text (e.g., a password), and dynamic text, which analyzes free-living text from participants [26]. Keystroke dynamics have largely been used for fraud detection and for authentication into computers or applications [26]. Touch dynamics, or touch gestures, evaluates touch strokes (size, length, speed, pressure, direction) and their corresponding coordinates on the touchscreen of a phone. Authentication using touch dynamics began as mobile devices were developed without a physical keyboard and rather a touchscreen [20]. Lastly, motion behavior authentication relies on the distinct movement patterns of individuals holding and interacting with a mobile device [27, 28].

Data produced by these sensors can be harnessed without additional hardware, evidenced by the growing body of research in the field of behavioral biometric authentication [21, 24, 29]. In child screen time research, employing continuous user identification may prove useful, especially when the device is being shared among a child and their family. Furthermore, applying behavioral biometric technology to screen time may be a relatively inexpensive solution, as it leverages built-in technology [24]. These benefits of behavioral biometrics are important attributes to consider when applying this technology to other contexts.

Behavioral biometric authentication is a highly established field of literature within cybersecurity; however, this technology has not yet been applied to objective screen time measurement research, to continuously identify the user of the mobile device [21, 30]. In order to begin applying this technology to screen time measurement, it is important to have an updated understanding of behavioral biometric technology and fit this updated understanding within the perspective of screen time research. The purpose of this scoping review was to first summarize the current state of behavioral biometric authentication, including identifying the behavioral biometric methods and data streams used, the characteristics predicted, and the model evaluation metrics used. This review also sought to characterize these findings within the scope of applying behavioral biometric technology to address the critical limitations of current measures of screen time to provide future directions for applying this technology to a public health context.

## Methods

This systematic review was conducted in accordance with the Preferred Reporting Items for Systematic Reviews and Meta-Analyses extension for Scoping Reviews (PRISMA-ScR) Checklist [31] and was pre-registered in the Open Science Framework database (10.17605/OSF.IO/92YCT).

### Information sources, search, and screening

**Fig. 1:**
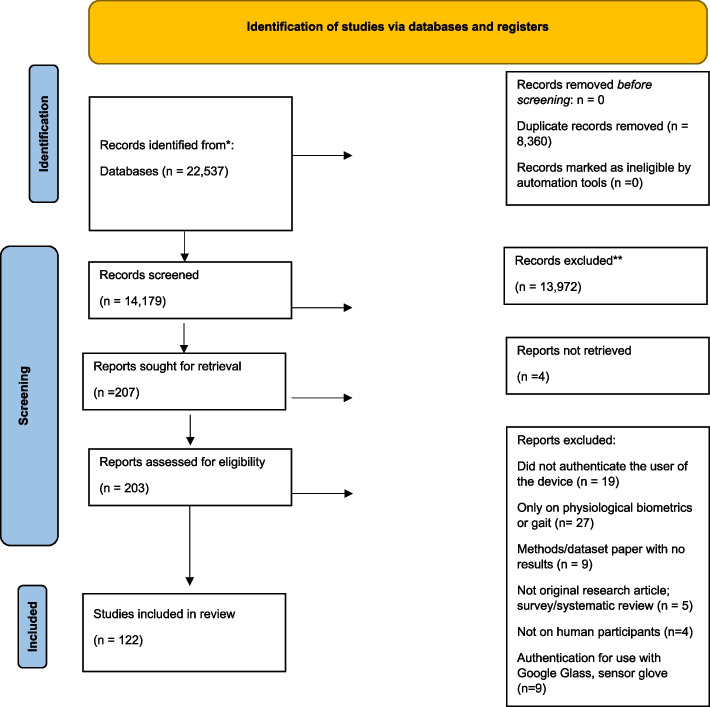
PRISMA flowchart

Literature searches were conducted in Web of Science Core Collection, Inspec in Engineering Village, Applied Science & Technology Source, IEEE Xplore, and PubMed, all of which were selected for their relevance to the topic and database size. The final database search was conducted on September 19, 2022. All authors and collaborators discussed the search strategy and the query strings specific to each database. Searches used keywords: smart device, tablet, phone, smartphone, handphone, mobile, Android, iOS, sensor, accelerometer, gyroscope, magnetometer, touch, biometric, hand, motion, move, swipe, keystroke, detect, verify, authenticate, infer, predict, determine, and classify, with Boolean operators, wildcard, and truncation used. The comprehensive list of search terms with notation specific to each database can be found in Additional file 2: Supplementary Table 2. The primary author (OF) performed the initial search. The search yielded 6,161 results from Web of Science Core Collection, 11,181 results from Inspec, 787 from Applied Science & Technology Source, 3584 from IEEE Xplore, and 823 from PubMed, for a total of 22,537 studies. References were exported to EndNote (Clarivate, London, UK), where an initial duplicate screen was completed using the “remove duplicates” function. Following this, references were exported to Covidence (Melbourne, Australia) for title and abstract screening, where duplicates were also removed, bringing the total studies for title and abstract screening down to 14,179. The primary author (OF) and an additional research assistant screened the titles and abstracts of the 14,179 studies on Covidence. Both reviewers established quality control of their screening process prior to independently screening the articles. This was done by screening 600 of the same articles independently and ensuring reviewers had consistency above 80%. Consistency between reviewers was met (99.9%) and then reviewers divided the remaining articles and independently screened the title and abstracts of those articles. Following title and abstract screening, 13,972 articles were excluded, and 207 articles were left for full text retrieval and screening. Four articles were not able to be located using the Interlibrary Loan (ILL) service; therefore, 203 articles were retrieved for eligibility assessment. The primary author (OF) reviewed the full texts of the 203 articles to assess whether these articles fully met the predefined inclusion and exclusion criteria. Of the 203 articles, 122 articles were considered eligible for inclusion and were extracted (Fig. 1).

### Eligibility criteria

Studies were required to focus on the sensors of mobile devices, defined as tablets or smartphones [32]. These sensors needed to be built-in to the device, including but not limited to motion sensors, accelerometer, gyroscope, magnetometer, and touch. Studies were eligible if they used these sensors for verification, detection, and/or authentication of the device user. Using an adapted version of the Meng 2015 [23] framework of biometric authentication, articles were required to focus on behavior profiling, keystroke dynamics (typing dynamics), touch dynamics, or motion behavior. Because the first smartphone (i.e., iPhone) was released in 2007 [33], and modern mobile tablets were developed after this in 2010, only articles after 2007 were included. Articles in a peer-reviewed academic source and published in English were eligible for inclusion. Articles that simulated data and did not collect data on human participants were excluded. Studies were excluded if they used other technology and/or required additional equipment beyond the mobile device (e.g., sensor glove, stylus) for verification. These articles were excluded because of their limited applicability to screen time measurement, as the goal of applying this technology would be to capture the typical way in which the child is interacting with their shared device. Articles that evaluated smart watches, fitness tracking devices, or wearable sensors were excluded. These articles were excluded because the tablet and smartphone are the preferred choice for digital media consumption in children [34] and are more closely related to health outcomes (e.g., sleep) [35]. Lastly, while the purpose of this review was to characterize these findings within the lens of child screen time measurement, we did not limit our search to only include studies on children. We included studies on adult populations since it is a relatively newer field and area of application to children and to inform future research on child populations from the current literature on adult biometric authentication.

### Data extraction

The primary author (OLF) extracted study characteristics (sample size, age, gender), data collection methods, data stream, model evaluation metrics, and performance of models from the 122 studies. All extracted data was reviewed by a second author (RGW).

#### Study characteristics

The extraction of study characteristics included details on the sample population, including sample size, gender distribution (number of female participants), and age (mean, standard deviation, and range). There were studies in this review that used freely available dataset(s) for their sample (*n* = 27), with several studies compiling data from more than one dataset (*n* = 8). Studies that used publicly available datasets for their sample are presented with a superscript letter in Table 1. Each superscript letter refers to a specific database, with several repeating databases used across studies, as depicted in Table 1. For studies using more than one dataset for their sample, sample sizes of the datasets were pooled, and number of female participants (gender distribution) were pooled. Additionally, for studies that used more than one dataset, we compiled the age ranges into one comprehensive age range across all included datasets. Device brand (iOS or Android) and outcome predicted (identity, age, or gender) were also extracted from the studies. The protocol of each study included was evaluated to determine whether it was a free-living or in-lab protocol. We defined in-lab protocols as those completed in a researcher-supervised controlled setting, while free-living refers to protocols in which participants use the device in their typical environment (e.g., home, work). Furthermore, we extracted whether the protocol was structured or free-use. We defined structured protocols as those in which the researchers give the participant a specific task to complete on the device, such as a questionnaire, a game, or using a particular application. Free-use protocols refer to protocols in which the participant can interact with the device in their normal manner and select which applications they use, with no restrictions from the researchers.

**Table 1:** Characteristics of included studies

Study Details		Sample population			Authentication		Study setting	
First author, year	Citation	Sample size	Female (*n*)	Age range	Device brand	Outcome predicted	Free-living vs. in-lab	Structured vs. free-use
Davis, 2020	[36]	48	21	< 40, > 40^*^	iOS	Sex, age	In-lab	Structured
Sun, 2016	[37]	19	8	18–35	Android	ID	In-lab	Structured
Liu, 2016	[38]	20	10	NR	Android	ID	In-lab	Structured
Maghsoudi, 2016	[39]	60	NR	NR	Android	ID	In-lab	Structured
Putri, 2016	[40]	29	NR	NR	Android	ID	In-lab	Structured
Lamiche, 2019	[41]	20	10	22–33	Android	ID	In-lab	Structured
Smith-Creasey, 2016	[42]	50	NR	NR	iOS	ID	In-lab	Free-use
Shih, 2015	[43]	10	4	22–30	Android	ID	In-lab	Structured
Zaidi, 2022^a,b,c,d^	[44]	350	NR	NR	Android	ID	In-lab	Structured
Soni, 2018	[45]	10	NR	NR	Android	ID	In-lab	Structured
Lin, 2012	[46]	20	4	18–40	Android	ID	In-lab	Structured
Li 2018	[47]	304	NR	NR	NR	ID	Free-living	Free-use
Smith-Creasey, 2019^e,f,g^	[48]	49	NR	NR	Android	ID	Free-living	Free-use
Salem, 2019	[49]	7	NR	NR	Android	ID	In-lab	Structured
Zhao, 2020	[50]	110	NR	NR	iOS	ID	In-lab	Structured
Qiao, 2015	[51]	10	NR	NR	Android	ID	In-lab	Structured
Smith-Creasey, 2019	[52]	20	NR	NR	Android	ID	In-lab	Structured
Alariki, 2016	[53]	18	NR	20–40	Android	ID	In-lab	Structured
Lee, 2017	[54]	12	NR	NR	Android	ID	In-lab	Structured
Li, 2020^h^	[55]	150	58	16–50	Android	ID	In-lab	Structured
Saini, 2020	[56]	40	NR	NR	NR	ID	In-lab	Free use
Takahashi, 2016	[57]	20	4	NR	Android	ID	In-lab	Structured
Deb, 2019	[58]	37	16	18–56	Android	ID	Free-living	Free-use
Leingang, 2018^i^	[59]	100	NR	NR	NR	ID	In-lab	Structured
Acien, 2019^j^	[60]	119	NR	3–6, < 25^*^	Android	ID	In-lab	Structured
Mahbub, 2016	[61]	48	NR	NR	Android	ID	Free-living	Free-use
Guarino, 2022	[62]	147	52	7–59	Android	Gender	In-lab	Structured
Wang, 2019^a^	[63]	21	NR	20–30	Android	ID	In-lab	Structured
Davarci, 2017	[64]	200	NR	3–11, 12–50	Android	Age	In-lab	Structured
Chakraborty, 2019^k^^,l,m^	[65]	60	NR	19–48	Android	ID	In-lab	Structured
Antal, 2015	[66]	42	18	20–46	Android	ID	In-lab	Structured
Roy, 2014^a^	[67]	41	13	10–69	Android	ID	In-lab	Structured
Salem, 2016	[68]	5	NR	NR	Android	ID	In-lab	Structured
Roy, 2019^a^	[69]	746	476	NR	Android	ID	In-lab	Structured
Lee, 2021	[70]	6	NR	NR	NR	Handedness	In-lab	Structured
Buriro, 2019	[71]	85	30	20–60	Android	ID	Free-living	Structured
Praher, 2016	[72]	8	NR	23–55	Android	ID	In-lab	Structured
Baran, 2019^n^	[73]	12	NR	NR	NR	ID	In-lab	Structured
Ali, 2016	[74]	6	3	NR	NR	ID	In-lab	Structured
Guerra-Casanova, 2012^o,p^	[75]	125	NR	NR	NR	ID	In-lab	Structured
Primo, 2017	[76]	27	NR	< 27^*^	Android	ID	In-lab	Structured
Yang, 2019	[77]	45	19	10–55	Android	ID	NR	NR
Wolff, 2013	[78]	6	NR	NR	NR	ID	Free-living	Free-use
Tse, 2019	[79]	31	NR	NR	NR	ID	In-lab	Structured
Antal, 2015^q,r,s^	[80]	120	54	20–49	Android	ID	In-lab	Structured
Laghari, 2016	[81]	10	NR	NR	Android	ID	In-lab	Structured
Tolosana, 2019	[82]	93	31	17–27^*^	Android	ID	In-lab	Structured
Ray, 2021	[83]	49	23	18–35 +	Android	ID	In-lab	Structured
Ambol, 2020	[84]	5	NR	NR	NR	ID	In-lab	Structured
Garbuz, 2019	[85]	36	NR	NR	NR	ID	In-lab	Structured
Dybczak, 2022	[86]	5	NR	NR	Android	ID	In-lab	Structured
Mumuria, 2015	[87]	73	NR	NR	Android	ID	In-lab	Free-use
Karanikiotis, 2020	[88]	2221	NR	NR	NR	ID	In-lab	Structured
Zhao, 2013	[89]	30	NR	NR	Android	ID	In-lab	Structured
Zhao, 2017	[90]	23	9	NR	Android	ID	Free-living	Free-use
Leyfer, 2019	[91]	14	NR	NR	Android	ID	Free-living	Free-use
Herath, 2022	[92]	3	NR	NR	NR	ID	NR	NR
Kumar, 2017	[93]	57	NR	NR	Android	ID	In-lab	Free-use
Barlas, 2020	[94]	30	11	NR	Android	ID	NR	NR
Incel, 2021	[95]	45	NR	18–42	Android	ID	In-lab	Structured
Hernandez-Ortega, 2017^j^	[96]	119	62	Children: 3–6, adults: < 25^*^	Android	ID	In-lab	Structured
Nguyen, 2017	[97]	20	6	20–30	Android	ID	In-lab	Structured
Al-Showarah, 2019	[98]	42	NR	Elderly: 60 + , younger: 20–39	Android	Age group	In-lab	Structured
Ng’ang’a, 2020	[99]	12	6	6 < 35, 6 < 35	NR	ID	In-lab	Structured
Ray-Dowling, 2022^i^	[100]	100	NR	NR	Android	ID	In-lab	Structured
Buriro, 2017	[101]	95	20	20–60	Android	ID	In-lab	Structured
Ouadjer, 2021^a^	[102]	41	13	10–69	Android	ID	Free-living	Free-use
Suharsono, 2020	[103]	50	NR	18–40	Android	ID	In-lab	Structured
Barra, 2018	[104]	38	NR	NR	Android	ID	In-lab	Structured
Mallet, 2022^i,t^	[105]	102	NR	NR	NR	ID	In-lab	Structured
Abate, 2019	[106]	100	NR	NR	Android	ID	In-lab	Structured
Cheng, 2020	[107]	100	41	Children: 3–17, adults: 18–59	Android	Age	In-lab	Structured
Alqarni, 2020	[108]	26	12	NR	Android	ID	In-lab	Structured
Rao, 2013	[109]	5	NR	NR	NR	ID	In-lab	Structured
Coakley, 2016	[110]	52	NR	NR	Android	ID	In-lab	Structured
Gautam, 2017	[111]	7	NR	NR	Android	ID	NR	NR
Deng, 2015^u^	[112]	55	NR	NR	Android	ID	In-lab	Structured
Roh, 2016	[113]	> 15^*^	NR	NR	Android	ID	In-lab	Structured
Acien, 2019^v^	[114]	48	12	22–31	NR	ID	Free-living	Free-use
Sun, 2021	[115]	26	17	30–63	NR	ID	Free-living	Free-use
Peralta, 2013	[116]	8	4	24–33	NR	ID	In-lab	Structured
Stragapede, 2022^w^	[117]	600	197	< 20–50	NR	ID	Free-living	Structured
Liang, 2020	[118]	20	12	10– > 59	Android	ID	Free-living	Free-use
Li, 2021	[119]	19	NR	NR	NR	ID	In-lab	Structured
Corpus, 2016	[120]	30	NR	NR	NR	ID	In-lab	Structured
Akhtar, 2017	[121]	150	NR	NR	Android	ID	Free-living	Structured
Song, 2017	[122]	161	26	18–55	Android	ID	In-lab	Structured
Primo, 2015	[123]	34	NR	< 25^*^	Android	ID	In-lab	Structured
Phillips, 2016	[124]	4	NR	NR	iOS	ID	In-lab	Structured
Li, 2016	[125]	42	NR	NR	Android	ID	In-lab	Structured
Haberfield, 2021	[126]	33	5	19–69	Android	ID	In-lab	Structured
Tharwat, 2019	[127]	51	25	NR	NR	ID	In-lab	Structured
Tang, 2022	[128]	10	NR	20–25	NR	ID	In-lab	Structured
Mahfouz, 2017	[129]	52	NR	NR	Android	ID	In-lab	Structured
Hernandez-Ortega, 2017^j^	[130]	119	62	Children: 3–6, adults: < 25	Android	Age	In-lab	Structured
Miguel-Hurtado, 2016^x^	[131]	116	59	18–35	Android	Sex	In-lab	Structured
Wang, 2020^i,v^	[132]	100	NR	20–30	NR	ID	NR	NR
Inguanez, 2016	[133]	32	10	NR	Android	ID	In-lab	Structured
Zhu, 2017	[134]	20	5	18–43	Android	ID	Free-living	Structured
Cheng, 2013	[135]	100	NR	NR	Android	ID	Free-living	Free-use
Gunn, 2018^i^	[136]	100	NR	NR	NR	ID	Free-living & in-lab	Free-use and structured
Wang, 2021	[137]	11	NR	NR	Android	ID	In-lab	Structured
Abate, 2016	[138]	100	NR	NR	Android	ID	In-lab	Structured
Acien, 2020^w^	[139]	600	197	< 20– > 50^*^	Android	ID	In-lab	Structured
Anusas-Amornkul, 2019	[140]	20	NR	NR	Android	ID	In-lab	Structured
Temper, 2016	[141]	25	9	19–65	Android	ID	Free-living	Structured
Roy, 2019	[142]	92	NR	7–65	Android	Age, Gender	In-lab	Structured
Shrestha, 2016	[143]	20	3	25–35	Android	ID	In-lab	Structured
Cascone, 2022^y,z,2^	[144]	243	148	7–65	Android	Gender, Age	In-lab	Structured
Temper, 2015	[145]	22	NR	15–60	Android	ID	In-lab	Structured
Frank, 2013	[146]	41	7	10–69	Android	ID	In-lab	Structured
Wantanabe, 2013	[147]	5	NR	NR	iOS	ID	In-lab	Structured
Volaka, 2019^i^	[148]	100	NR	NR	Android	ID	In-lab	Structured
Brown, 2020	[149]	1	NR	NR	Android	ID	In-lab	Structured
Sharma, 2017	[150]	42	NR	NR	Android	ID	In-lab	Free-use
Kroeze, 2016	[151]	30	NR	NR	Android	ID	In-lab	Free-use
Filippov, 2018	[152]	21	NR	NR	NR	ID	In-lab	Free-use
Karakaya, 2019^i^	[153]	100	NR	NR	NR	ID	In-lab	Structured
Serwadda, 2013	[154]	190	NR	NR	Android	ID	In-lab	Structured
Buriro, 2016	[155]	30	8	NR	Android	ID	In-lab	NR
Shen, 2016	[156]	48	19	18–50	Android	ID	In-lab	Structured
Stylios, 2022	[157]	39	NR	NR	Android	ID	In-lab	Structured

*NR* not reported

^a^Frank dataset

^b^Serwadda dataset

^c^Antal dataset

^d^Mabhub dataset

^e^SHR dataset

^f^MSC dataset

^g^GCU dataset

^h^Article encompassed two studies

^i^H-MOG dataset

^j^Vatavu dataset

^k^UCAI-HAR dataset

^l^UT-Data-Complex dataset

^m^shoiab dataset

^n^gesture dataset

^o^GBS2GestureDB1database

^p^GB2SGestureDB2 database

^q^Dataset_11f

^r^Dataset_8f

^s^Dataset_3f

^t^BioIdent dataset

^u^Stanford TapDynamics dataset

^v^UMDAA-02 dataset

^w^HuMIdb dataset

^x^SSD dataset

^y^RHU dataset

^z^KDAp dataset

^*^Study did not provide further details on age range

^2^TDAS dataset

#### Methods

Extraction of study characteristics also included identifying the biometric method(s) employed for authentication of the user and/or detection of demographic characteristics. We first recorded the biometric method described by each study in the precise language used by the authors. Given the lack of standardized terminology in the field of biometric authentication, these methods needed to be condensed into broader categories. The categories for biometric methods were consolidated into four categories, with agreement from all authors. These categories included *movement* (encompassing hand movement, arm gesture, hand gesture, and posture), *behavior profiling*, *keystroke dynamics*, and *touch gestures*.

#### Data stream

Extraction of study characteristics also included the identification of the specific data stream(s), or sensors, used for biometric authentication. We first recorded the data stream(s) used in each study using the precise language used by the authors. Similar to categorizing biometric methods, the categories for data streams also needed to be condensed to broader categories of similar characteristics. These categories included *accelerometer* (gravity, linear acceleration), *orientation*, *gyroscope* (rotation, angular velocity), *touch*, *location*, *magnetometer*, and *other* (ambient light, Bluetooth, temperature, proximity, application usage, power).

#### Model evaluation metrics

Extraction of study characteristics also included identifying the model evaluation metric(s) used in each study. We first identified the evaluation metric described in each study using the precise language used by the authors. Metrics were condensed into broader categories given the lack of consistent terminology in machine learning model performance metrics. These categories included *area under the curve* (receiver operating characteristic), *equal error rate* (EER), *precision*, *recall* (sensitivity, true acceptance rate, true positive rate), *false rejection rate* (FRR, false negative rate), *false acceptance rate* (FAR, false positive rate, “false alarm rate”), *accuracy* (correct recognition rate, mean recognition rate, success rate), *F1 score* (F-measure), and *other* (kappa, root mean square error H-mean, detection error tradeoff curve, specificity/true rejection rate, average match rate, mean square error rate, average number of impostor actions, and average number of genuine actions).

### Quality assessment

The quality of the included studies was assessed using an adapted framework from Papi 2017 [158], which is a research quality scale specific to the field of engineering with a focus on sensor technology (Additional file 1: Supplementary Table 1). The primary author (OLF) assessed the study quality of all 122 studies. Each question was scored as either 1, meeting the criteria, or 0, not meeting the criteria. Composite quality assessment scores were calculated by adding together the number of criteria met, with a score of 14 meaning that the study was of highest quality and a score of 0 meaning that the study was of lowest quality.

### Data analysis

The characteristics of the included studies were tabulated in Excel (Microsoft, Version 2304). We then compiled summary statistics in Excel to describe our findings. Means and standard deviations were calculated for sample size and gender distribution across all studies.

## Results

### Study characteristics

Across all 122 studies, sample sizes ranged from 1 to 2221 participants, with an average of 89 participants per study (± 224.2). Android was the most common operating system, with 89 studies (73%) using Android devices for their protocol(s). The iOS operating system was used in 5 (4%) of protocols and the remaining 28 studies (23%) did not report the operating system used. Most of the studies (*n* = 112, 92%) identified the specific user of the device, while 5 (4%) studies aimed to detect the gender of the user and 7 studies (6%) aimed to detect the age/age group of the user. For the study setting, most study protocols were conducted in a lab setting (*n* = 99, 81%), while fewer studies were carried out in a free-living environment (*n* = 17, 14%), one study used both lab and free-living settings, and 5 studies (4%) did not provide sufficient information to determine study setting. Most protocols were structured (*n* = 96, 79%), with specific guidance and directions given to the participants on how to interact with the device (e.g., playing a game, watching a specific video). Few studies (*n* = 19, 16%) allowed participants to interact with the device in their normal manner, considered “free use” of the device, one study had both structured and free-use, and 6 studies (5%) did not provide sufficient information to determine protocol format. Many studies did not report demographic characteristics of the sample; 75 (61%) did not report gender, 70 (57%) did not report age, and none reported race/ethnicity. Of those that did report gender, on average, the distribution of female participants was 39% of the sample. Of the 122 studies, 75 studies (61%) did not report an age range, 34 studies (28%) had a sample of adults, and 13 studies (11%) had age ranges that included children (< 18 years).

### Methods

Of the 122 studies included in this review, 63 (52%) used movement (e.g., hand movement, hand or arm gesture and posture) as their biometric method for authentication. Thirty studies (25%) used keystroke dynamics for biometric authentication, while 76 studies (62%) used touch gestures. Behavior profiling, such as app usage, battery, and WiFi, was used in 7 studies (6%) for biometric authentication.

### Data stream

Touch was the most extensively used data stream, with 93 studies using touch behavior for biometric authentication. The accelerometer sensor was the second most frequently used sensor of this review, with *n* = 68 studies (56%). Other data streams employed include gyroscope (*n* = 46 studies, 38%), orientation (*n* = 9 studies, 7%), location (*n* = 8 studies, 7%), and magnetometer (*n* = 22 studies, 18%). As depicted in Additional file 3: Supplementary Table 3, all other data streams that were used in less than 3 studies were combined into an “Other” category. These included ambient light (*n* = 3, 2%), Bluetooth (*n* = 3, 2%), temperature (*n* = 1), proximity (*n* = 3, 2%), application usage (*n* = 1), power/battery level (*n* = 2), motion quaternion (*n* = 1), directional heading (*n* = 1), and heat map (*n* = 1).

### Model evaluation metrics

When evaluating the performance of their models, the included studies used a wide range of evaluation metrics. Equal error rate (EER) and accuracy were the most highly used evaluation metrics, with 57 studies (47%) using EER and 56 studies (46%) using accuracy. Following EER and accuracy, false rejection rate (FRR) (*n* = 42 studies, 34%) and false acceptance rate (FAR) (*n* = 47 studies, 39%) were also highly used to evaluate model performance. Area under the curve (AUC) and the receiver operating characteristic curve (ROC) were used in 20 studies (16%). Recall/sensitivity (*n* = 20, 16%), F1 score (*n* = 14, 11%), and precision (*n* = 10, 8%) were also frequently used among the included studies. As depicted in Additional file 3: Supplementary Table 3, all other model evaluation metrics that were used in less than 4 studies were combined into an “Other” category. These included kappa (*n* = 2), root mean square error (RMSE) (*n* = 1), H-mean (*n* = 1), detection error tradeoff (DET) curve (*n* = 4, 3%), specificity/true rejection rate (*n* = 3), average match rate (*n* = 1), mean square error rate (*n* = 1), average number of impostor actions (ANIA) (*n* = 2), and average number of genuine actions (ANGIA/ANGA) (*n* = 2).

### Quality of the included studies

The average quality score of the included studies was 5.5 out of 14, with a high score of 11 and a low score of 3. The most commonly met criteria were #11, reporting main outcomes, with 122 out of 122 studies meeting this criterion, and #1, clearly stating research objectives, with 121 out of 122 studies meeting this criterion. Most studies also met the criteria for #12, reporting the main findings (*n* = 119 studies), and for #13, clearly describing and justifying the statistical tests (*n* = 118 studies). The selection of sensors (#9) was appropriately justified in 65 studies, while data handling was clearly described (#10) in 35 studies. Only some studies met the criteria for #14, clearly describing the limitations (*n* = 33), or met the criteria for #8, clearly describing the equipment design (*n* = 23). Few studies of this review met the criteria for #3, adequately describing the study population (*n* = 19), as many did not report demographic characteristics such as age and gender. Only 8 out of the 122 studies met the criteria for #5, appropriately describing the sampling methodology, and only 7 out of the 122 studies met the criteria for #7, providing detailed methods that could be replicated. None of the included studies met the criteria for #4, specifying eligibility criteria, and for #6, providing a rationale for the sample size.

## Discussion

Behavioral biometrics have the potential to improve screen time measurement because researchers can capitalize on built-in mobile device sensors to determine who is using the device at specific time points to address a critical limitation in child screen time research. This scoping review sought to summarize the current state of behavioral biometric authentication, including identifying the behavioral biometric methods used, the data streams used, the characteristics predicted, and the model evaluation metrics used. On a larger scale, this updated understanding of the methodology of behavioral biometric studies can inform future research applying this technology to a public health context.

Overall, in the 122 included studies, the most highly used behavioral biometric methods were touch gestures and movement. The most highly used data streams for behavioral biometric authentication were touch and accelerometry. Motion sensors, such as accelerometer, gyroscope, and magnetometer, are straightforward to access and record with a sensor tracking application (e.g., Sensor Log) on mobile devices. Using touch sensors presents more challenges, both in terms of accessing this sensor stream as well as the privacy and security concerns of participants. Several of the studies using touch in this review used their own gaming application that only tracked touch behavior while the participant was using the application, which has limited applicability to screen time measurement, as it only records touch behavior during the use of one application. In addition to challenges in accessing this sensor stream, there are privacy concerns, as research participants may not feel comfortable with sensor data from their devices being collected continuously. While collecting motion behavior may not be as much of a concern, there may be a particular concern in tracking touch sensor data when using banking applications or typing passwords (e.g., concerns in researchers deciphering passwords). Therefore, while touch is a highly used behavioral biometric method, it may have more limited applicability to screen time measurement when compared to motion sensors (e.g., accelerometer, gyroscope, magnetometer).

Most behavioral biometric authentication studies in this review aimed to identify the user of the device, with fewer studies aiming to detect demographic characteristics, such as age and gender. Studies that used behavioral biometrics to detect age were designed to tailor technology interfaces towards children (e.g., widget layout) and to improve parental control options. Similarly, in studies examining the ability of behavioral biometrics to determine gender, their objective was to adapt interfaces to be more relevant for the user. Based on current evidence, behavioral biometrics are less accurate at detecting demographic characteristics compared to detecting a unique user [159]. It is likely more challenging to identify similar characteristics in user behavior across a group of individuals, as user interaction can vary substantially on an inter-individual level [60, 159]. Relative to applied screen time measurement in a public health context, detecting the age of the user may be a relevant finding to distinguish between the parent and the child when they are sharing the device. However, the ability to detect only the age of a user would not be as useful when a child shares a device with a sibling of a similar age. Thus, determining the unique identity of a user of the device rather than demographic characteristics would be more relevant for research purposes.

Furthermore, of the included studies, a majority of studies used samples of adult participants, with fewer studies tested on samples of children. The lack of research on children highlights a gap in the literature, as there are inherent behavioral differences in the ways in which children interact with mobile devices compared to adults [159] (e.g., children are more active), and findings from adult studies cannot be universally applied to children. Therefore, we need additional research on biometrics among children before applying this technology to measure children’s screen time.

The most popular model evaluation metrics used in the included studies were equal error rate (EER), accuracy, false acceptance rate (FAR), and false rejection rate (FRR). There were a wide range of model evaluation metrics used across studies, with several reporting the same metric under different terms. For example, several studies used the term “Correct Recognition Rate,” instead of accuracy and “False Positive Rate” instead of false acceptance rate. This highlights a lack of standardization in terminology that is consistent across the field of behavioral biometric authentication, which limits our ability to compare findings across studies.

Of the studies included in this review, the average study quality was low (5.5/14), highlighting the lack of proper reporting in many of the studies in the field of behavioral biometric authentication. Overall, most authors did not provide sufficient information on equipment design, study population, sampling methodology, and eligibility criteria. Very few authors provided adequate justification for their sample size. The insufficiency in reporting key elements of study design limits the ability to replicate these findings in other samples and contexts. Furthermore, the lack of standardization in the terminology used across studies hampers the ability to make larger conclusions on the efficacy of behavioral biometrics and their application in the measurement of children’s screen time.

### Behavioral biometric tools and innovative directions for future research

Though the purpose of this review is to examine the current scope of literature on behavioral biometrics through the lens of its application to public health (i.e., screen time measurement), it is necessary to also distinguish this from the domain of behavioral biometrics research for security. Given the vast amount of sensitive information stored on mobile devices, secure user authentication has become a prominent concern and a highly studied concept over recent years [22, 160, 161]. User authentication has shifted from “what you know,” such as an ID, PIN, or password, to “what you are,” or biometric authentication, with behavioral biometrics referring to the specific user-device interaction. A specific framework developed by Bo and colleagues in 2013, SilentSense, provides a touch-based biometrics model that leverages touch events from the system API [162, 163]. This tool additionally integrated movement into its scheme, presenting a multi-modal authentication method. Another more recent development in behavioral biometrics is the generation of behavioral biometric datasets using engaging tools [164, 165]. There have been challenges in collecting biometric data on participants due to the long protocols necessary to capture sufficient amount of data [164]. Therefore, researchers have developed gaming applications that collect a variety of behavioral biometric data (e.g., keystroke dynamics, touch gestures, motion) [164, 165]. BrainRun, developed by Papamichail and colleagues, is a cognitive skills gaming application that collects touch data. BioGames, developed by Stylios and colleagues, is also a gaming application that collects touch, motion, and keystroke data [164]. These applications are important tools in the feasible generation of large-scale behavioral biometric data. Lastly, a challenge within behavioral biometrics research is the power usage concerns on mobile devices, particularly for continuous authentication methods. In future behavioral biometrics research, power consumption of individual applications should be monitored to ensure that the authentication application is not substantially impacting the device battery. Power consumption of individual applications can be monitored using a method from Murmuria and colleagues that uses per-subsystem time shares from the operating system, which can provide clarity on the feasibility of deploying behavioral biometric methods in a larger research context [166].

### Advantages and disadvantages of the behavioral biometric methods

As these behavioral biometric methods have been highly studied and applied for use within the field of cybersecurity, this work has highlighted some of the advantages and disadvantages associated with each of these methods. While all methods are subject to privacy concerns [30], behavior profiling in particular has been scrutinized for its reliance on sensitive and private data (e.g., calls, texts, location). However, an advantage of behavior profiling is that unlike other methods (e.g., keystroke dynamics), it does not require the user to perform a specific activity for authentication [25]. A disadvantage of keystroke dynamics is that its accuracy for user authentication can be impacted by factors including injury, psychological state (e.g., stress), and distraction [167]. Additionally, the way in which an individual types on a keyboard is considered less permanent than other traits, such as physiological biometrics (e.g., facial and fingerprint recognition) [167]. However, relative to other authentication methods, keystroke dynamics is relatively low cost and does not rely on external hardware. Additionally, the way in which an individual types is challenging to replicate; therefore, this method can detect impostors more effectively [167]. An advantage of touch dynamics authentication is that the user does not need to complete a specific task for authentication; rather, this method works continuously in the background [20]. However, a disadvantage could be identifying the most salient features for user authentication, as using a large number of touch features increases data size and subsequently can slow down authentication speed [23]. Lastly, motion authentication can be impacted by behavioral variability, as this type of authentication is reliant on the user to interact with the device similarly over time [168]. However, similar to other methods, motion authentication can be an unobtrusive authentication method [168], and there may be less privacy concerns compared to touch-based authentication.

### Methodological considerations and implications for future research

Subsequent research should examine the effectiveness of behavioral biometrics to determine the user of the device among children across development. Most of the studies included in this review exclusively used adult samples, which has limited applicability to child screen time research. The present review also highlighted the lack of studies being done on iOS devices (iPhone, iPad) in the field of behavioral biometrics. This is a limitation of the field because iOS use is highly prevalent, as 55% of tablets in the USA are iPads [169]. In 2022, over 50% of smartphone owners in the USA used an iPhone, surpassing Android for the first time in history [170]. A majority of the studies (*n* = 85) tested Android devices, with only 5 studies using an iOS operating system, warranting further testing on a diversity of devices, including both iOS and Android.

When applying this technology to objective screen time measurement, participants may be apprehensive about researchers tracking mobile device usage data. However, there are practices in place to reduce concerns with tracking technology. Specifically with the passive-sensing application Chronicle, data are not associated with IP addresses or phone numbers and only indicate the type of application used (e.g., educational, social media), not the information on websites visited or the content of messages and emails. Parents are comfortable with using passive sensing technology when participating in a research study, as indicated by a feasibility study reporting no dropouts due to privacy concerns in using this technology [171]. While passive sensing applications have been shown to be accepted for use by families, future research can examine the extent to which families are comfortable with sensor tracking technology (e.g., accelerometer, gyroscope, touch) continuously monitoring user behavior on shared mobile devices. Prior to employing this technology in screen time measurement on a large scale, a necessary first step is to determine the feasibility and acceptability of this technology for families participating in research.

Additionally, research using this technology to measure screen time should consider the storage and battery life concerns inherent to using mobile device usage data. The computational burden of running applications to track sensor data may impact the feasibility of longitudinally monitoring screen time behavior in children [30]. Selecting the appropriate sensor tracking application and sampling frequency to use, as well as only recording sensor data when the device is unlocked must be a priority for researchers using behavioral biometric technology for screen time research [159].

Lastly, within the field of behavioral biometric authentication, there is a necessity to standardize the terminology used to describe various elements of behavioral biometrics. The lack of uniform language needs to be addressed to apply this technology on a larger scale. A way in which the field of behavioral biometrics can move towards more cohesive language is by adopting best-practice guidelines for reporting performance metrics, similar to the fields of physical activity measurement [172] and sleep measurement [173].

The present review has several strengths, including a comprehensive review of the current state of behavioral biometric authentication. This provided an updated evaluation of the most highly used behavioral biometric methods, data streams, and model evaluation metrics. The current review is limited by the low quality of the included studies and the lack of consistency in the terminology used across studies. Given the lack of standardization in model evaluation metrics, we were unable to sum results across studies and use meta-analytic methods to evaluate the overall efficacy of behavioral biometrics in identifying the user of a device. Furthermore, a limitation of the current review is the narrow focus on behavioral biometrics (touch, accelerometry, behavioral profiling) and not including studies on physiological biometrics. While physiological biometrics presents an important tool in authentication, these sensors (e.g., camera, video) are not freely available and feasible to use in public health research. Despite these limitations, behavioral biometric technology highlights a window of opportunity, as it shows the initial potential to harness sensor data to identify the user of a device. This review can inform future research applying behavioral biometric technology to contexts outside of cybersecurity and to address the limitations of objective measures of screen time.

### Supplementary Information

**Additional file 1: Supplementary Table 1.** Quality Assessment Framework.

**Additional file 2: Supplementary Table 2.** List of Search Terms.

**Additional file 3: Supplementary Table 3.** Data Stream, Biometric Methods & Model Evaluation Metrics of Included Studies.

**Additional file 4: Table 2.** Quality Assessment of Included Studies.

## Data Availability

Not applicable; no datasets were generated or analyzed during the current study.
