# Supplementary material for: The utility of behavioral biometrics in user authentication and demographic characteristic detection: a scoping review
Source: Syst Rev. 2024 Feb 8;13:61. doi: 10.1186/s13643-024-02451-1 (PMC10851515; doi:10.1186/s13643-024-02451-1)
Supplement: Supplementary file 1 — Additional file 1: Supplementary Table 1. Quality Assessment Framework. [file 13643_2024_2451_MOESM1_ESM.pdf]

SUPPLEMENTARY/ADDITIONAL FILES

Supplementary Table 1. Quality Assessment Framework

Criterion	Description
**1**	Were the research objectives or aims clearly stated?
**2**	Was the study design clearly described?
**3**	Was the study population adequately described?
**4**	Were the eligibility criteria specified?
**5**	Was the sampling methodology appropriately described?
**6**	Was the sample size used justified?
**7**	Did the method description enable accurate replication of the measurement procedures?
**8**	Was the equipment design and set up clearly described?
**9**	Were sensors used justified?
**10**	Was the signal/data handling described?
**11**	Were the main outcomes measured and the related calculations (if applicable) clearly described?
**12**	Were the main findings of the study stated?
**13**	Were the statistical tests clearly described and justified? Machine Learning
**14**	Were limitations of the study clearly described?
