# Supplementary material for: The utility of behavioral biometrics in user authentication and demographic characteristic detection: a scoping review
Source: Syst Rev. 2024 Feb 8;13:61. doi: 10.1186/s13643-024-02451-1 (PMC10851515; doi:10.1186/s13643-024-02451-1)
Supplement: Supplementary file 2 — Additional file 2: Supplementary Table 2. List of Search Terms. [file 13643_2024_2451_MOESM2_ESM.pdf]

Supplementary Table 2. List of Search Terms

Database Search				
**Database**	**Search Terms**	**Year**	**# Studies**	**Date Searched**
IEEE Xplore	("Abstract":"smart device" OR "Abstract":tablet OR "Abstract":phone OR "Abstract":smartphone OR "Abstract":handphone OR "Abstract":mobile OR "Abstract":Android OR "Abstract":IOS) AND ("Abstract":sensor OR "Abstract":accelerometer OR "Abstract":gyroscope OR "Abstract":magnetometer OR "Abstract":touch) AND ("Abstract":biometric* OR "Abstract":hand OR "Abstract":motion OR "Abstract":finger OR "Abstract":voice OR "Abstract":move* OR "Abstract":swipe OR "Abstract":keystroke OR "Abstract":face) AND ("Abstract":detect* OR "Abstract":verif* OR "Abstract":authentica* OR "Abstract":infer* OR "Abstract":predict* OR "Abstract":determin* OR "Abstract":classif*)	2007 - 2022	3,584	9/14/2022
Web of Science Core Collection	(((AB=("smart device" OR tablet OR phone OR smartphone OR handphone OR mobile OR Android OR IOS)) AND AB=(sensor OR accelerometer OR gyroscope OR magnetometer OR touch)) AND AB=(biometric* OR hand OR motion OR eye OR finger OR voice OR move* OR swipe OR keystroke OR face)) AND AB=(detect* OR verif* OR authentica* OR infer* OR predict* OR determin* OR classif*)	01-01-2007 - 09-14-2022	6,161	9/14/2022

Inspec in Engineering Village	((((("smart device" OR tablet OR phone OR smartphone OR handphone OR mobile OR Android OR IOS) WN KY) AND ((sensor OR accelerometer OR gyroscope OR magnetometer OR touch) WN KY)) AND ((biometric* OR hand OR motion OR eye OR finger OR voice OR move* OR swipe OR keystroke OR face) WN KY)) AND ((detect* OR verif* OR authentica* OR infer* OR predict* OR determin* OR classif*) WN KY))	2007-2022	11,181	9/19/2022
Applied Science & Technology Source	AB ("smart device" OR tablet OR phone OR smartphone OR handphone OR mobile OR Android OR IOS) AND AB (sensor OR accelerometer OR gyroscope OR magnetometer OR touch) AND AB (biometric* OR hand OR motion OR eye OR finger OR voice OR move* OR swipe OR keystroke OR face) AND AB (detect* OR verif* OR authentica* OR infer* OR predict* OR determin* OR classif*)	2007 - 2022	787	9/14/2022
PubMed	((("smart device"[Title/Abstract] OR tablet[Title/Abstract] OR phone[Title/Abstract] OR smartphone[Title/Abstract] OR handphone[Title/Abstract] OR mobile[Title/Abstract] OR Android[Title/Abstract] OR IOS[Title/Abstract]) AND (sensor[Title/Abstract] OR accelerometer[Title/Abstract] OR gyroscope[Title/Abstract] OR magnetometer[Title/Abstract] OR touch[Title/Abstract])) AND (biometric*[Title/Abstract] OR hand[Title/Abstract] OR motion[Title/Abstract] OR eye[Title/Abstract] OR finger[Title/Abstract] OR voice[Title/Abstract] OR move*[Title/Abstract] OR swipe[Title/Abstract] OR keystroke[Title/Abstract] OR face[Title/Abstract])) AND (detect*[Title/Abstract] OR verif*[Title/Abstract] OR authentica*[Title/Abstract] OR infer*[Title/Abstract] OR predict*[Title/Abstract] OR determin*[Title/Abstract] OR classif*[Title/Abstract])	2007 - 2022	823	9/14/2022
