# Supplementary material for: The utility of behavioral biometrics in user authentication and demographic characteristic detection: a scoping review
Source: Syst Rev. 2024 Feb 8;13:61. doi: 10.1186/s13643-024-02451-1 (PMC10851515; doi:10.1186/s13643-024-02451-1)
Supplement: Supplementary file 3 — Additional file 3: Supplementary Table 3. Data Stream, Biometric Methods & Model Evaluation Metrics of Included Studies. [file 13643_2024_2451_MOESM3_ESM.docx]

SUPPLEMENTARY/ADDITIONAL FILES

**Supplementary Table 3.** Data Stream, Biometric Methods & Model Evaluation Metrics of Included Studies

| Study Details | Biometric Methods | | | | Model Evaluation Metrics | | | | | | | | | Data Streams | | | | | | |
| --- | --- | --- | --- | --- | --- | --- | --- | --- | --- | --- | --- | --- | --- | --- | --- | --- | --- | --- | --- | --- |
| First Author, Year | Move | BehavProf | KSD | Touch | AUC/  ROC | EER | Precision | Recall | FRR | FAR | Acc | F1 | Other | Accel | Orient | Gyroscope | Touch | Loc | Mag | Other |
| Davis, 2020 | 0 | 0 | 0 | 1 | 0 | 0 | 1 | 1 | 0 | 0 | 1 | 0 | 0 | 0 | 0 | 0 | 1 | 0 | 0 |  |
| Sun, 2016 | 1 | 0 | 0 | 0 | 0 | 0 | 0 | 0 | 1 | 1 | 0  0 | 0 | 0 | 1 | 0 | 0 | 0 | 0 | 0 |  |
| Liu, 2016 | 1 | 0 | 0 | 0 | 0 | 0 | 0 | 0 | 1 | 1 | 0  0 | 0 | 0 | 1 | 1 | 0 | 0 | 0 | 0 |  |
| Maghsoudi, 2016 | 1 | 0 | 0 | 0 | 0 | 0 | 0 | 0 | 0 | 0 | 1 | 0 | 0 | 1 | 0 | 1 | 0 | 0 | 0 |  |
| Putri, 2016 | 0 | 0 | 1 | 1 | 0 | 0 | 0 | 0 | 1 | 1 | 1 | 0 | 0 | 0 | 0 | 0 | 1 | 0 | 0 |  |
| Lamiche, 2019 | 0 | 0 | 1 | 0 | 0 | 1 | 0 | 1 | 0 | 0 | 1 | 0 | 0 | 1 | 0 | 0 | 1 | 0 | 0 |  |
| Smith-Creasey, 2016 | 0 | 0 | 0 | 1 | 0 | 1 | 0 | 0 | 0 | 1 | 0  0 | 0 | 0 | 0 | 0 | 0 | 1 | 0 | 0 |  |
| Shih, 2015 | 1 | 0 | 0 | 1 | 0 | 0 | 1 | 1 | 1 | 1 | 1 | 1 | 0 | 1 | 0 | 0 | 1 | 0 | 0 |  |
| Zaidi, 2022 | 0 | 0 | 0 | 1 | 0 | 1 | 0 | 0 | 0 | 0 | 0  0 | 0 | 0 | 0 | 0 | 0 | 1 | 0 | 0 |  |
| Soni, 2018 | 0 | 0 | 0 | 1 | 0 | 0 | 0 | 0 | 0 | 0 | 1 | 0 | 0 | 0 | 0 | 0 | 1 | 0 | 0 |  |
| Lin, 2012 | 1 | 0 | 0 | 0 | 0 | 1 | 0 | 0 | 1 | 1 | 0  0 | 0 | 0 | 0 | 1 | 0 | 0 | 0 | 0 |  |
| Li 2018 | 1 | 0 | 0 | 0 | 0 | 1 | 0 | 0 | 0 | 0 | 0  0 | 0 | 0 | 1 | 0 | 1 | 0 | 0 | 0 |  |
| Smith-Creasey, 2019 | 1 | 1 | 0 | 0 | 1 | 1 | 0 | 0 | 1 | 1 | 0  0 | 0 | 0 | 1 | 0 | 1 | 0 | 1 | 1 |  |
| Salem, 2019 | 0 | 0 | 1 | 0 | 0 | 1 | 0 | 0 | 1 | 1 | 0  0 | 0 | 0 | 0 | 0 | 0 | 1 | 0 | 0 |  |
| Zhao, 2020 | 1 | 0 | 0 | 0 | 1 | 0 | 0 | 0 | 0 | 0 | 1 | 0 | 0 | 1 | 0 | 1 | 0 | 0 | 1 |  |
| Qiao, 2015 | 0 | 0 | 0 | 1 | 0 | 0 | 0 | 0 | 1 | 1 | 1 | 0 | 0 | 0 | 0 | 0 | 1 | 0 | 0 |  |
| Smith-Creasey, 2019 | 1 | 0 | 0 | 1 | 1 | 1 | 0 | 0 | 1 | 1 | 0  0 | 0 | 0 | 1 | 0 | 1 | 1 | 0 | 0 |  |
| Alariki, 2016 | 0 | 0 | 0 | 1 | 0 | 0 | 0 | 0 | 0 | 0 | 1 | 0 | 0 | 0 | 0 | 0 | 1 | 0 | 0 |  |
| Lee, 2017 | 1 | 0 | 1 | 0 | 0 | 0 | 0 | 0 | 0 | 0 | 1 | 0 | 0 | 1 | 0 | 1 | 1 | 0 | 0 |  |
| Li, 2020 | 0 | 0 | 0 | 1 | 0 | 0 | 0 | 0 | 1 | 1 | 0  0 | 0 | 0 | 0 | 0 | 0 | 1 | 0 | 0 |  |
| Saini, 2020 | 0 | 0 | 1 | 0 | 0 | 1 | 0 | 0 | 1 | 1 | 1 | 0 | 0 | 1 | 0 | 0 | 1 | 0 | 0 |  |
| Takahashi, 2016 | 1 | 0 | 1 | 0 | 0 | 1 | 0 | 0 | 0 | 0 | 0  0 | 0 | 0 | 1 | 1 | 0 | 1 | 0 | 0 |  |
| Deb, 2019 | 1 | 1 | 1 | 0 | 0 | 0 | 0 |  | 0 | 1 | 0 | 0 | 0 | 1 | 0 | 1 | 1 | 1 | 1 |  |
| Leingang, 2018 | 0 | 0 | 0 | 1 | 0 | 0 | 0 | 0 | 0 | 0 | 1 | 0 | 0 | 0 | 0 | 0 | 1 | 0 | 0 |  |
| Acien, 2019 | 0 | 0 | 0 | 1 | 0 | 0 | 0 | 0 | 0 | 0 | 1 | 0 | 0 | 0 | 0 | 0 | 1 | 0 | 0 |  |
| Mahbub, 2016 | 1 | 1 | 0 | 1 | 0 | 0 | 0 | 0 | 0 | 0 | 1 | 1 | 0 | 1 | 0 | 1 | 1 | 1 | 1 |  |
| Guarino, 2022 | 0 | 0 | 0 | 1 | 0 | 0 | 0 | 0 | 0 | 0 | 0  0 | 1 | 0 | 0 | 0 | 0 | 1 | 0 | 0 |  |
| Wang, 2019 | 1 | 0 | 0 | 0 | 1 | 0 | 0 | 0 | 1 | 1 | 0  0 | 0 | 0 | 1 | 0 | 1 | 0 | 0 | 1 |  |
| Davarci, 2017 | 1 | 0 | 0 | 1 | 0 | 0 | 1 | 1 | 0 | 0 | 1 | 1 | 0 | 1 | 0 | 0 | 1 | 0 | 0 |  |
| Chakraborty, 2019 | 1 | 0 | 0 | 0 | 0 | 0 | 1 | 1 | 0 | 1 | 0  0 | 1 | 0 | 1 | 0 | 1 | 0 | 0 | 1 |  |
| Antal, 2015 | 0 | 0 | 1 | 0 | 1 | 1 | 0 | 0 | 0 | 0 | 0  0 | 0 | 1 | 1 | 0 | 0 | 1 | 0 | 0 |  |
| Roy, 2014 | 0 | 0 | 0 | 1 | 0 | 1 | 0 | 0 | 1 | 1 | 0  0 | 0 | 0 | 0 | 0 | 0 | 1 | 0 | 0 |  |
| Salem, 2016 | 0 | 0 | 1 | 0 | 0 | 1 | 0 | 0 | 1 | 1 | 0  0 | 0 | 0 | 0 | 0 | 0 | 1 | 0 | 0 |  |
| Roy, 2019 | 0 | 0 | 1 | 0 | 0 | 1 | 0 | 0 | 0 | 0 | 1 | 0 | 0 | 0 | 0 | 0 | 1 | 0 | 0 |  |
| Lee, 2021 | 1 | 0 | 0 | 1 | 0 | 0 | 0 | 0 | 0 | 0 | 0  0 | 0 | 1 | 1 | 0 | 1 | 1 | 0 | 0 |  |
| Buriro, 2019 | 1 | 0 | 0 | 1 | 0 | 0 | 0 |  | 0 | 0 | 0 | 0 | 0 | 1 | 0 | 1 | 1 | 0 | 1 |  |
| Praher, 2016 | 0 | 0 | 1 | 0 | 0 | 1 | 0 | 0 | 1 | 1 | 0  0 | 0 | 0 | 0 | 0 | 0 | 1 | 0 | 0 |  |
| Baran, 2019 | 0 | 0 | 0 | 1 | 0 | 0 | 0 | 0 | 0 | 0 | 1 | 0 | 0 | 0 | 0 | 0 | 1 | 0 | 0 |  |
| Ali, 2016 | 0 | 0 | 0 | 1 | 0 | 0 | 0 | 0 | 0 | 0 | 1 | 0 | 0 | 1 | 0 | 0 | 1 | 0 | 0 |  |
| Guerra-Casanova, 2012 | 1 | 0 | 0 | 0 | 0 | 1 | 0 | 0 | 1 | 1 | 0  0 | 0 | 0 | 1 | 0 | 0 | 0 | 0 | 0 |  |
| Primo, 2017 | 0 | 0 | 1 | 0 | 1 | 1 | 0 | 0 | 0 | 0 | 0  0 | 0 | 0 | 0 | 0 | 0 | 1 | 0 | 0 |  |
| Yang, 2019 | 0 | 0 | 0 | 1 | 0 | 0 | 1 | 1 | 0 | 0 | 1 | 1 | 0 | 0 | 0 | 0 | 1 | 0 | 0 |  |
| Wolff, 2013 | 1 | 0 | 1 | 1 | 0 | 0 | 0 | 0 | 0 | 0 | 1 | 0 | 0 | 1 | 0 | 0 | 1 | 0 | 0 |  |
| Tse, 2019 | 0 | 0 | 1 | 1 | 0 | 0 | 0 | 0 | 0 | 0 | 1 | 1 | 0 | 0 | 0 | 0 | 1 | 0 | 0 |  |
| Antal, 2015, | 1 | 0 | 0 | 1 | 0 | 1 | 0 | 0 | 0 | 0 | 0  0 | 0 | 1 | 1 | 0 | 0 | 1 | 0 | 0 |  |
| Laghari, 2016 | 1 | 0 | 0 | 0 | 0 | 0 | 0 | 0 | 1 | 1 | 0  0 | 0 | 0 | 1 | 0 | 0 | 0 | 0 | 0 |  |
| Tolosana, 2019 | 0 | 0 | 0 | 1 | 0 | 1 | 0 | 0 | 0 | 0 | 0  0 | 0 | 0 | 0 | 0 | 0 | 1 | 0 | 0 |  |
| Ray, 2021 | 1 | 0 | 1 | 1 | 0 | 1 | 0 | 0 | 0 | 0 | 0  0 | 0 | 0 | 1 | 0 | 1 | 1 | 0 | 0 |  |
| Ambol, 2020 | 1 | 0 | 0 | 0 | 1 | 0 | 1 | 1 | 0 | 0 | 1 | 1 | 0 | 1 | 0 | 1 | 0 | 0 | 1 |  |
| Garbuz, 2019 | 0 | 0 | 0 | 1 | 0 | 0 | 0 | 0 | 1 | 1 | 0  0 | 0 | 1 | 1 | 0 | 1 | 1 | 0 | 0 |  |
| Dybczak, 2022 | 1 | 0 | 0 | 0 | 0 | 0 | 0 | 0 | 0 | 0 | 1 | 0 | 0 | 1 | 0 | 1 | 0 | 0 | 0 |  |
| Mumuria, 2015 | 0 | 0 | 0 | 1 | 1 | 1 | 0 | 0 | 1 | 1 | 0  0 | 0 | 0 | 1 | 0 | 1 | 0 | 0 | 0 |  |
| Karanikiotis, 2020 | 0 | 0 | 0 | 1 | 0 | 0 | 0 | 0 | 1 | 1 | 0  0 | 0 | 0 | 0 | 0 | 0 | 1 | 0 | 0 |  |
| Zhao, 2013 | 0 | 0 | 0 | 1 | 0 | 1 | 0 | 0 | 0 | 0 | 0  0 | 0 | 0 | 0 | 0 | 0 | 1 | 0 | 0 |  |
| Zhao, 2017 | 0 | 0 | 0 | 1 | 0 | 0 | 0 | 0 | 1 | 1 | 0  0 | 0 | 0 | 0 | 0 | 0 | 1 | 0 | 0 |  |
| Leyfer, 2019 | 0 | 0 | 0 | 1 | 1 | 0 | 0 | 0 | 0 | 0 | 0  0 | 0 | 0 | 0 | 0 | 0 | 1 | 0 | 0 |  |
| Herath, 2022 | 0 | 0 | 1 | 0 | 0 | 0 | 0 | 0 | 1 | 1 | 1 | 1 | 0 | 0 | 0 | 0 | 1 | 0 | 0 |  |
| Kumar, 2017 | 1 | 0 | 0 | 0 | 0 | 1 | 0 | 0 | 0 | 0 | 0  0 | 0 | 0 | 1 | 0 | 0 | 0 | 0 | 0 |  |
| Barlas, 2020 | 1 | 0 | 0 | 1 | 0 | 1 | 0 |  | 0 | 1 | 0 | 0 | 0 | 1 | 0 | 1 | 1 | 0 | 1 |  |
| Incel, 2021 | 1 | 0 | 0 | 1 | 0 | 1 | 0 |  | 1 | 1 | 1  1 | 0 | 0 | 1 | 0 | 1 | 1 | 0 | 1 |  |
| Hernandez-Ortega, 2017 | 0 | 0 | 0 | 1 | 0 | 0 | 0 | 0 | 0 | 0 | 1 | 0 | 0 | 0 | 0 | 0 | 1 | 0 | 0 |  |
| Nguyen, 2017 | 0 | 0 | 0 | 1 | 0 | 1 | 0 | 0 | 0 | 0 | 0  0 | 0 | 0 | 0 | 0 | 0 | 1 | 0 | 0 |  |
| Al-Showarah, 2019 | 0 | 0 | 0 | 1 | 0 | 0 | 0 | 0 | 0 | 0 | 1 | 0 | 0 | 0 | 0 | 0 | 1 | 0 | 0 |  |
| Ng’ang’a, 2020 | 0 | 0 | 0 | 1 | 0 | 0 | 0 | 0 | 1 | 1 | 1 | 0 | 0 | 0 | 0 | 0 | 1 | 0 | 0 |  |
| Ray-Dowling, 2022 | 1 | 0 | 0 | 1 | 0 | 1 | 0 | 0 | 0 | 0 | 0  0 | 0 | 0 | 1 | 0 | 1 | 1 | 0 | 0 |  |
| Buriro, 2017 | 1 | 0 | 0 | 1 | 1 | 0 | 0 |  | 1 | 1 | 0 | 0 | 1 | 1 | 1 | 1 | 1 | 0 | 1 |  |
| Ouadjer, 2021 | 0 | 0 | 0 | 1 | 0 | 0 | 0 | 0 | 0 | 0 | 1 | 1 | 1 | 0 | 0 | 0 | 1 | 0 | 0 |  |
| Suharsono, 2020 | 1 | 0 | 0 | 1 | 0 | 1 | 0 | 0 | 0 | 0 | 0  0 | 0 | 0 | 0 | 1 | 0 | 1 | 0 | 0 |  |
| Barra, 2018 | 1 | 0 | 0 | 0 | 1 | 1 | 0 | 0 | 0 | 0 | 1 | 0 | 0 | 1 | 0 | 1 | 0 | 1 | 0 |  |
| Mallet, 2022 | 1 | 0 | 0 | 1 | 0 | 1 | 1 | 1 | 0 | 0 | 1 | 1 | 0 | 1 | 0 | 1 | 1 | 0 | 1 |  |
| Abate, 2019 | 1 | 0 | 0 | 0 | 1 | 1 | 0 | 0 | 0 | 0 | 0  0 | 0 | 0 | 1 | 0 | 1 | 0 | 0 | 0 |  |
| Cheng, 2020 | 1 | 0 | 0 | 1 | 1 | 1 | 0 |  | 0 | 1 | 0 | 0 | 0 | 1 | 0 | 1 | 1 | 0 | 0 |  |
| Alqarni, 2020 | 1 | 0 | 0 | 0 | 0 | 0 | 0 | 0 | 0 | 0 | 1 | 1 | 1 | 1 | 0 | 1 | 0 | 0 | 0 |  |
| Rao, 2013 | 1 | 0 | 0 | 1 | 0 | 0 | 0 | 0 | 1 | 1 | 0  0 | 0 | 0 | 1 | 0 | 0 | 1 | 0 | 0 |  |
| Coakley, 2016 | 1 | 0 | 1 | 0 | 1 | 1 | 0 | 0 | 0 | 0 | 0  0 | 0 | 0 | 1 | 0 | 1 | 1 | 0 | 0 |  |
| Gautam, 2017 | 0 | 0 | 1 | 0 | 0 | 1 | 0 | 0 | 1 | 1 | 0  0 | 0 | 0 | 0 | 0 | 0 | 1 | 0 | 0 |  |
| Deng, 2015 | 1 | 0 | 1 | 0 | 0 | 1 | 0 | 0 | 1 | 1 | 0  0 | 0 | 0 | 1 | 0 | 0 | 1 | 0 | 0 |  |
| Roh, 2016 | 1 | 0 | 1 | 0 | 0 | 1 | 0 | 0 | 0 | 0 | 0  0 | 0 | 0 | 1 | 0 | 0 | 1 | 0 | 0 |  |
| Acien, 2019 | 0 | 1 | 1 | 0 | 0 | 1 | 0 | 0 | 0 | 0 | 0  0 | 0 | 0 | 0 | 0 | 1 | 1 | 1 | 1 |  |
| Sun, 2021 | 1 | 0 | 0 | 1 | 0 | 1 | 0 | 0 | 0 | 0 | 1 | 0 | 1 | 1 | 0 | 0 | 1 | 0 | 0 |  |
| Peralta, 2013 | 0 | 0 | 0 | 1 | 0 | 0 | 0 | 0 | 0 | 0 | 1 | 0 | 0 | 0 | 0 | 0 | 1 | 0 | 0 |  |
| Stragapede, 2022 | 1 | 0 | 0 | 1 | 0 | 1 | 0 | 0 | 0 | 0 | 0  0 | 0 | 1 | 1 | 0 | 1 | 0 | 0 | 1 |  |
| Liang, 2020 | 1 | 0 | 0 | 1 | 0 | 0 | 0 | 0 | 1 | 1 | 1 | 0 | 0 | 1 | 0 | 1 | 1 | 0 | 0 |  |
| Li, 2021 | 1 | 0 | 0 | 0 | 0 | 0 | 0 | 0 | 1 | 1 | 1 | 0 | 0 | 1 | 0 | 1 | 0 | 0 | 0 |  |
| Corpus, 2016 | 1 | 0 | 1 | 0 | 0 | 0 | 0 | 0 | 1 | 1 | 1 | 0 | 0 | 1 | 0 | 0 | 1 | 0 | 0 |  |
| Akhtar, 2017 | 1 | 0 | 0 | 1 | 0 | 1 | 0 |  | 0 | 0 | 0 | 0 | 0 | 1 | 1 | 1 | 0 | 0 | 1 |  |
| Song, 2017 | 0 | 0 | 0 | 1 | 0 | 1 | 0 | 0 | 0 | 0 | 0  0 | 0 | 0 | 0 | 0 | 0 | 1 | 0 | 0 |  |
| Primo, 2015 | 0 | 0 | 0 | 1 | 0 | 1 | 0 | 0 | 0 | 0 | 1 | 0 | 0 | 0 | 0 | 0 | 1 | 0 | 0 |  |
| Phillips, 2016 | 1 | 1 | 0 | 0 | 0 | 0 | 0 | 0 | 0 | 0 | 1 | 0 | 0 | 1 | 0 | 1 | 0 | 1 | 0 |  |
| Li, 2016 | 0 | 0 | 0 | 1 | 0 | 1 | 0 | 0 | 1 | 1 | 0  0 | 0 | 0 | 0 | 0 | 0 | 1 | 0 | 0 |  |
| Haberfield, 2021 | 0 | 0 | 0 | 1 | 1 | 1 | 0 | 0 | 0 | 0 | 0  0 | 0 | 0 | 0 | 0 | 0 | 1 | 0 | 0 |  |
| Tharwat, 2019 | 0 | 0 | 1 | 0 | 0 | 0 | 0 | 0 | 0 | 0 | 1 | 0 | 0 | 0 | 0 | 0 | 1 | 0 | 0 |  |
| Tang, 2022 | 0 | 0 | 1 | 0 | 0 | 0 | 0 | 0 | 0 | 0 | 1 | 0 | 0 | 0 | 0 | 0 | 1 | 0 | 0 |  |
| Mahfouz, 2017 | 0 | 0 | 1 | 1 | 1 | 1 | 0 | 0 | 0 | 0 | 0  0 | 0 | 0 | 0 | 0 | 0 | 1 | 0 | 0 |  |
| Hernandez-Ortega, 2017 | 0 | 0 | 0 | 1 | 0 | 0 | 0 | 0 | 0 | 0 | 1 | 0 | 0 | 0 | 0 | 0 | 1 | 0 | 0 |  |
| Miguel-Hurtado, 2016 | 0 | 0 | 0 | 1 | 0 | 0 | 0 | 0 | 0 | 0 | 1 | 0 | 0 | 0 | 0 | 0 | 1 | 0 | 0 |  |
| Wang, 2020 | 1 | 0 | 0 | 0 | 1 | 0 | 0 | 0 | 1 | 1 | 0  0 | 0 | 0 | 1 | 0 | 1 | 0 | 0 | 1 |  |
| Inguanez, 2016 | 0 | 0 | 0 | 1 | 0 | 0 | 1 | 1 | 1 | 1 | 1 | 0 | 0 | 0 | 0 | 0 | 0 | 0 | 0 |  |
| Zhu, 2017 | 1 | 0 | 0 | 0 | 0 | 1 | 0 | 0 | 1 | 1 | 0  0 | 0 | 0 | 1 | 0 | 1 | 0 | 0 | 0 |  |
| Cheng, 2013 | 1 | 0 | 0 | 1 | 0 | 0 | 0 | 0 | 0 | 0 | 1 | 0 | 0 | 1 | 0 | 1 | 1 | 0 | 0 |  |
| Gunn, 2018 | 0 | 0 | 0 | 1 | 0 | 0 | 0 | 0 | 0 | 0 | 1 | 0 | 1 | 1 | 0 | 1 | 1 | 0 | 1 |  |
| Wang, 2021 | 1 | 0 | 0 | 0 | 0 | 0 | 0 | 0 | 1 | 0 | 0  0 | 0 | 0 | 1 | 1 | 1 | 0 | 0 | 1 |  |
| Abate, 2016 | 1 | 0 | 0 | 0 | 1 | 1 | 0 | 0 | 1 | 1 | 0  0 | 0 | 0 | 1 | 0 | 1 | 0 | 0 | 0 |  |
| Acien, 2020 | 1 | 1 | 1 | 1 | 0 | 1 | 0 | 0 | 0 | 0 | 1 | 0 | 0 | 1 | 1 | 1 | 1 | 1 | 1 |  |
| Anusas-Amornkul, 2019 | 1 | 0 | 1 | 0 | 0 | 1 | 0 | 0 | 0 | 0 | 1 | 0 | 0 | 1 | 0 | 0 | 1 | 0 | 0 |  |
| Temper, 2016 | 0 | 0 | 1 | 1 | 0 | 0 | 0 | 0 | 0 | 0 | 1 | 0 | 0 | 0 | 0 | 0 | 1 | 0 | 0 |  |
| Roy, 2019 | 0 | 0 | 1 | 0 | 1 | 0 | 0 | 1 | 0 | 0 | 1 | 0 | 1 | 0 | 0 | 0 | 1 | 0 | 0 |  |
| Shrestha, 2016 | 1 | 0 | 0 | 1 | 0 | 0 | 1 | 1 | 0 | 0 | 0  0 | 1 | 0 | 1 | 1 | 1 | 0 | 0 | 1 |  |
| Cascone, 2022 | 0 | 0 | 0 | 1 | 0 | 0 | 0 | 0 | 0 | 0 | 1 | 0 | 0 | 0 | 0 | 0 | 1 | 0 | 0 |  |
| Temper, 2015 | 1 | 0 | 0 | 1 | 0 | 1 | 0 | 0 | 0 | 0 | 0  0 | 0 | 0 | 1 | 0 | 0 | 1 | 0 | 0 |  |
| Frank, 2013 | 0 | 0 | 0 | 1 | 0 | 1 | 0 | 0 | 0 | 0 | 0  0 | 0 | 0 | 0 | 0 | 0 | 1 | 0 | 0 |  |
| Wantanabe, 2013 | 1 | 0 | 0 | 1 | 0 | 0 | 0 | 0 | 1 | 1 | 0  0 | 0 | 0 | 1 | 0 | 0 | 1 | 0 | 0 |  |
| Volaka, 2019 | 1 | 0 | 0 | 1 | 0 | 1 | 1 | 0 | 0 | 0 | 1 | 1 | 0 | 1 | 0 | 1 | 1 | 0 | 0 |  |
| Brown, 2020 | 1 | 0 | 0 | 0 | 0 | 0 | 0 | 0 | 0 | 0 | 1 | 0 | 0 | 1 | 0 | 1 | 0 | 0 | 0 |  |
| Sharma, 2017 | 0 | 0 | 0 | 1 | 0 | 1 | 0 | 0 | 0 | 0 | 1 | 0 | 0 | 0 | 0 | 0 | 1 | 0 | 0 |  |
| Kroeze, 2016 | 0 | 0 | 0 | 1 | 1 | 1 | 0 | 0 | 1 | 1 | 0  0 | 0 | 0 | 0 | 0 | 0 | 1 | 0 | 0 |  |
| Filippov, 2018 | 0 | 0 | 0 | 1 | 0 | 0 | 0 | 0 | 1 | 1 | 0  0 | 0 | 1 | 0 | 0 | 0 | 1 | 0 | 0 |  |
| Karakaya, 2019 | 1 | 0 | 0 | 1 | 0 | 0 | 0 | 0 | 0 | 0 |  | 0 | 0 | 1 | 0 | 1 | 1 | 0 | 1 |  |
| Serwadda, 2013 | 0 | 0 | 0 | 1 | 0 | 1 | 0 | 0 | 0 | 0 | 0  0 | 0 | 0 | 0 | 0 | 0 | 1 | 0 | 0 |  |
| Buriro, 2016 |  |  |  |  |  |  |  |  |  |  |  |  |  |  |  |  |  |  |  |  |
| Shen, 2016 |  |  |  |  |  |  |  |  |  |  |  |  |  |  |  |  |  |  |  |  |
| Stylios, 2022 |  |  |  |  |  |  |  |  |  |  |  |  |  |  |  |  |  |  |  |  |
| Total | 63 | 7 | 30 | 76 | 20 | 57 | 10 | 20 | 42 | 47 | 56 | 14 | 13 | 68 | 9 | 46 | 93 | 8 | 22 | 9 |
| Move = Movement, Behav Prof = Behavioral Profiling, KSD  = keystroke dynamics; AUC = Area Under the Curve; ROC = Receiver Operating Characteristics; EER = Equal Error Rate; FRR = False Rejection Rate; FAR = False Acceptance Rate; FPR = False Positive Rate; TAR = True Acceptance Rate, TPR = True Positive Rate; Acc = accuracy; Accel = accelerometer; Orient = orientation; loc = location; mag = magnetometer; Other = Kappa, Root Mean Square Error H-mean, Detection Error Tradeoff curve, True Rejection Rate, Average Match Rate, Mean Square Error Rate, specificity, Average Number of Impostor Actions, and Average Number of Genuine Actions  **Color Coding:** Light Blue = Method/stream/metric not used in study, Dark Blue = Method/stream/metric used in study | | | | | | | | | | | | | | | | | | | | |
